# Supplementary material for: Selective conversion of syngas to C4+ long-chain alcohols
Source: Nat Commun. 2026 Mar 23;17:4323. doi: 10.1038/s41467-026-70994-z (PMC13172505; doi:10.1038/s41467-026-70994-z)
Supplement: Supplementary file 1 — Supplementary Information [file 41467_2026_70994_MOESM1_ESM.pdf]

Supplementary Information

Selective conversion of syngas to C_{4+} long-chain alcohols

Yihui Li,[1,†] Ziang Zhao,[1,†] Miao Jiang,[1] Guoqing Wang,[1] Zheng Li,[1,2] Wei Lu,[1] Wenhao, Cui,[1] Rong Liu,[4] Ronghe Lin,[3,*] Yu Meng,[4,*] Yuan Lyu,[1] Li Yan,[1,*] Hejun Zhu,[1,*]& Yunjie Ding[1,3,*]

[1] State Key Laboratory of Catalysis, Dalian Institute of Chemical Physics, Chinese Academy of Sciences, Dalian, 116023, China

[2] University of Chinese Academy of Sciences, Beijing, 100049, China

[3] Key Laboratory of the Ministry of Education for Advanced Catalysis Materials, Zhejiang Key Laboratory of Advanced Catalysis and Adsorption Materials, Hangzhou Institute of Advanced Studies, Zhejiang Normal University, 1108 Gengwen Road, Hangzhou 311231, China

[4] Shaanxi Key Laboratory of Low Metamorphic Coal Clean Utilization, School of Chemistry and Chemical Engineering, Yulin University, Yulin, 719000, China

[†] These authors contributed equally to this work.

[*]Corresponding authors: catalysis.lin@zjnu.edu.cn; mengyu@yulinu.edu.cn yanli@dicp.ac.cn; zhuhj@dicp.ac.cn; dyj@dicp.ac.cn.

Table S1. The catalytic performances of syngas-to-oxygenates/olefins catalysts for the syngas conversion.

Catalysts	GHSV / h^{-1}	H_2/CO	*T* / °C	X_{CO} / %	*S* / %					*Alkanes/ Olefins*	$C_{3+}^{=}/C^{=}$ / %	C_{4+}*Oxy./Oxy.* / %	*S* ($C_{3+}^{=}$ & C_{4+}*Oxy.*) / %
					CH_4	CO_2	C_{2+}	$C_{2+}^{=}$	Oxy				
Co/C	2000	2.0	210	21.7	22.0	0.4	34.3	24.7	18.5	2.3	98.9	70.3	37.4
$Co_{0.1}Cs/C$	2000	2.0	210	11.7	8.4	2.1	23.9	36.4	29.2	0.9	90.0	77.5	55.4
$Co_{0.5}Mn/C$	2000	2.0	210	20.0	10.2	0.6	29.4	35.8	24.0	1.1	93.0	69.7	50.0
$Co_{0.5}Mn_{0.1}Cs/C$	2000	2.0	210	29.2	6.6	2.7	20.7	31.5	38.6	0.9	94.6	85.5	65.6

Reaction conditions: H_2/CO = 2, *T* = 210 °C and *P* = 3 MPa.

Catalysts	GHSV / h^{-1}	H_2/CO	*T* / °C	X_{CO} / %	*S* / %					*Alkanes/ Olefins*	$C_{3+}^{=}/C^{=}$ / %	C_{4+}*Oxy./Oxy.* / %	*S* ($C_{3+}^{=}$ & C_{4+}*Oxy.*) / %
					CH_4	CO_2	C_{2+}	$C_{2+}^{=}$	Oxy				
$Co_{0.5}Mn_{0.1}Cs/C$	2000	2.0	210	29.2	6.6	2.7	20.7	31.5	38.6	0.9	94.6	85.5	65.6
$Co_{0.5}Mn_{0.1}Cs/C$	2000	1.5	210	25.5	5.6	2.6	20.5	37.6	33.6	0.7	96.4	93.3	68.4
$Co_{0.5}Mn_{0.1}Cs/C$	4000	1.5	210	12.4	2.2	0.6	20.0	30.4	46.4	0.7	96.0	93.1	72.5

Reaction conditions: *T* = 210 °C and *P* = 3 MPa.

Table S2. Catalytic performances of various catalysts for syngas conversion to oxygenates in the literatures.

Catalysts	GHSV / h^{-1}	H_2/CO	*T* / °C	X_{CO} / %	*S* / %				*Alk/Ole* / %	*Refs.*
					CO_2	Alk	Ole	Oxy		
Co/C	2000	2.0	210	21.7	0.4	56.3	24.7	18.5	2.3	TW
$Co_{0.1}Cs/C$	2000	2.0	210	11.7	2.1	32.3	36.4	29.2	0.9	TW
$Co_{0.5}Mn/C$	2000	2.0	210	20.0	0.6	39.6	35.8	24.0	1.1	TW
Co_1Mn/C	2000	2.0	210	18.9	1.0	37.9	41.0	20.2	0.9	TW
$Co_{0.5}Mn_{0.1}Cs/C$	2000	2.0	210	29.2	2.7	27.3	31.5	38.6	0.9	TW
$Co_{0.5}Mn_{0.1}Cs/C$	2000	1.5	210	25.5	2.6	26.1	37.6	33.6	0.7	TW
$Co_{0.5}Mn_{0.1}Cs/C$	4000	1.5	210	12.4	0.6	22.2	30.4	46.4	0.7	TW
Co_4Cu_1	7200[a]	1.5	240	11.0	1.0	53.2	15.0	30.8	3.5	[1]
Co_1Cu_1	2000	2	240	5.0	12.0	35.0	25.0	28.0	1.4	[2]
$Co_1Cu_2Nb_{0.2}$	40[b]	1.5	200	6.0	1.3	28.3	18.4	52.0	1.5	[3]
$Co_1Cu_1Mn_1$	2000	2	240	18.0	8.0	34.0	24.0	34.0	1.4	[2]
$Co_4Mn_1K_{0.1}$	40[b]	1.5	220	18.0	16.0	24.0	20.0	38.0	1.2	[4]
0.3Rh-CoMn	2000[a]	1	220	29.5	20.4	24.6	26.7	28.3	0.9	[5]
0.3Ru-CoMn	2000[a]	1	220	32.6	23.3	33.7	20.0	22.9	1.7	[5]
Co/MnOx@quasi-MOF-74	4500[a]	2	230	21.4	0.8	38.6	21.6	35.9	1.8	[6]

[a] mL $gcat^{-1}$ h^{-1}. [b] ml min^{-1}.

Table S3. The detailed E, H, S, ZPE, G values for each IS, TS, and FS states of the reaction pathway studied in different calculation models

Model	Adsorbate	E	ZPE	H	S	G
Co	$*CCH_2C_3H_7+*H$	-474.95	3.534	4.019	0.989	3.030
	TS	-474.27	3.489	3.960	0.956	3.004
	$*CHCH_2C_3H_7+*$	-474.278	3.698	4.219	1.061	3.158
	TS	-472.448	3.370	3.881	1.088	2.793
	$*CHCHC_3H_7+*H$	-474.094	3.462	3.967	1.060	2.907
	TS	-472.934	3.730	4.211	0.988	3.223
	$*CH_2CHC_3H_7+*$	-472.941	3.893	4.377	1.000	3.377
	$*CHCH_2C_3H_7+*H$	-478.162	3.698	4.219	1.061	3.158
	TS	-477.592	3.730	4.211	0.988	3.223
	$*CH_2CH_2C_3H_7+*$	-477.964	3.893	4.377	1.000	3.377
	$*CH_2CH_2C_3H_7+*H$	-481.88	4.083	4.621	1.127	3.495
	TS	-480.83	4.037	4.586	1.170	3.416
	$*CH_3CH_2C_3H_7+*$	-482.229	4.259	4.777	1.144	3.633
	$*CHCH_2C_3H_7+*CO$	-490.867	3.677	4.292	1.265	3.027
	TS	-489.777	3.737	4.344	1.274	3.070
	$*COCHCH_2C_3H_7+*$	-490.13	3.798	4.411	1.287	3.123
Co_2C-Co	$*CCH_2C_3H_7+*H$	-969.528	3.513	4.006	1.036	2.970
	TS	-968.492	3.470	3.962	1.013	2.949
	$*CHCH_2C_3H_7+*$	-968.527	3.581	4.076	1.028	3.047
	TS	-967.788	3.405	3.917	1.078	2.839
	$*CHCHC_3H_7+*H$	-968.934	3.458	3.944	0.964	2.980
	TS	-967.887	3.389	3.848	0.912	2.936
	$*CH_2CHC_3H_7+*$	-968.642	3.604	4.097	1.028	3.070
	$*CHCH_2C_3H_7+*H$	-972.649	3.754	4.271	1.046	3.225
	TS	-972.067	3.767	4.256	0.985	3.271
	$*CH_2CH_2C_3H_7+*$	-972.188	3.891	4.398	1.054	3.345
	$*CH_2CH_2C_3H_7+*H$	-976.258	4.072	4.613	1.126	3.487
	TS	-975.168	4.094	4.577	0.983	3.593
	$*CH_3CH_2C_3H_7+*$	-976.757	4.255	4.698	0.912	3.786
	$*CHCH_2C_3H_7+*CO$	-985.418	3.768	4.400	1.300	3.100
	TS	-984.556	3.728	4.349	1.271	3.079
	$*COCHCH_2C_3H_7+*$	-984.819	3.779	4.373	1.203	3.170
Cs_2O-Co_2C-Co	$*CCH_2C_3H_7+*H$	-983.122	3.528	4.017	0.995	3.023
	TS	-982.226	3.477	3.967	1.008	2.959
	$*CHCH_2C_3H_7+*$	-982.362	3.588	4.077	1.013	3.065
	TS	-981.784	3.400	3.867	0.945	2.922
	$*CHCHC_3H_7+*H$	-982.744	3.477	3.963	0.988	2.975
	TS	-982.001	3.463	3.927	0.934	2.993
	$*CH_2CHC_3H_7+*$	-982.265	3.591	4.078	0.995	3.084
	$*CHCH_2C_3H_7+*H$	-986.395	3.767	4.281	1.034	3.247
	TS	-985.455	3.762	4.259	1.022	3.237

	$*CH_2CH_2C_3H_7+*$	-985.83	3.874	4.380	1.054	3.326
	$*CH_2CH_2C_3H_7+*H$	-989.829	4.048	4.584	1.105	3.480
	TS	-988.487	4.056	4.559	1.075	3.483
	$*CH_3CH_2C_3H_7+*$	-990.342	4.266	4.786	1.142	3.644
	$*CHCH_2C_3H_7+*CO$	-999.288	3.752	4.377	1.280	3.097
	TS	-998.468	3.711	4.340	1.301	3.039
	$*COCHCH_2C_3H_7+*$	-998.548	3.751	4.353	1.199	3.154
Cu	$*C_5H_{11}CHO+*H$	-529.36	4.661801	5.155494	1.065767	4.089727
	TS	-527.71	4.618257	5.08528	0.955278	4.130002
	$*C_5H_{11}CH_2O+*$	-528.93	4.764797	5.170733	0.800561	4.370172
	$*C_5H_{11}CH_2O+*H$	-532.276	4.9056	5.351431	0.91401	4.43742
	TS	-532.106	4.87405	5.357289	0.99015	4.367139
	$*C_5H_{11}CH_2OH+*$	-533.395	5.117547	5.586337	0.96158	4.624758
Cu/ZrO_2	$*C_5H_{11}CHO+*H$	-1839.46	4.650485	5.047186	0.777926	4.26926
	TS	-1838.82	4.618898	5.04363	0.872195	4.171435
	$*C_5H_{11}CH_2O+*$	-1839.38	4.786809	5.199093	0.85693	4.342163
	$*C_5H_{11}CH_2O+*H$	-1842.54	4.970997	5.416286	0.913088	4.503198
	TS	-1842.08	4.931647	5.352619	0.848944	4.503675
	$*C_5H_{11}CH_2OH+*$	-1843.15	5.147832	5.560021	0.828798	4.731223

Table S4. The catalytic performances of different combinations of catalysts in the tandem system for the syngas-to-oxygenates/olefins conversion.

Catalysts	X_{CO} / %	*S* / %						*n*/*i* *mol mol*$^{-1}$	C_{3+}=/C= / %	C_{4+}Oxy./ Oxy. / %
		CH_4	CO_2	C_{2+}	$C_{2+}^{=}$	aldehydes	alcohols			
CoMnCs/C|Rh/3v-POPs-PPh_3	22.9	3.8	3.0	13.2	0.9	34.4	44.6	5.1	22.2	54.4
CoMnCs/C|Rh/POPs-BP&PPh_3	23.5	4.6	1.7	18.7	0.7	33.1	41.2	4.0	100.0	69.2
CoMnCs/C|$CuZrO_2/SiO_2$	24.8	5.1	1.9	21.3	30.4	1.1	40.1	-	90.1	81.8
CoMnCs/C|CuZnAl	30.0	3.5	13.8	22.0	21.8	0.3	38.6	-	94.5	86.1
CoMnCs/C|Cu/Al_2O_3	26.0	3.9	22.7	25.4	10.8	0.6	36.6	-	95.4	62.6
CoMnCs/C|Cu/SiO_2	23.2	1.8	20.7	22.6	8.6	1.0	45.4	-	100.0	79.1
CoMnCs/C|RuPd/C	22.5	3.5	1.0	20.6	22.3	7.4	45.2	-	90.6	69.0
CoMnCs/C|Ru/C	21.4	4.6	1.7	25.1	16.1	7.0	45.5	-	89.4	62.7
CoMnCs/C|Pt/TiO_2	18.1	1.9	0.8	20.7	34.5	13.1	29.0	-	95.7	91.2
CoMnCs/C|PtSn/TiO_2	22.7	4.2	6.5	15.8	32.9	0.4	40.1	-	89.1	86.7

Reaction conditions: H_2/CO = 1.5, F = 65 ml min^{-1}, T = 210 °C (syngas-to-oxygenates/olefins), T = 140 °C (hydroformylation/hydrogenation), and P = 3 MPa.

Table S5. Catalytic performances of various catalysts for syngas conversion to oxygenates in the literatures.

Catalysts	*T* / °C	*P* / MPa	H_2/CO / mol mol^{-1}	WHSV/ ml g^{-1} h^{-1}	X_{CO} / %	S_{CO2} / %	S_{OH} / %	S_{C2+OH} / %	S_{C4+OH} / %	S_{C6+OH} / %	STY_{C4+OH} / g kg_{cat}^{-1} h^{-1}	refs.
**CoMnCs/C|Rh/3v-POPs-PPh$_3$|CuZrO$_2$/SiO$_2$**	210/140 [a]	3.0	1.5	1600 [b]	14.7	1.7	80.8	80.0	73.0	35.7	70.8	this work
	210/140 [a]	8.0	1.5	1600 [b]	17.2	1.4	86.5	86.3	79.9	45.5	89.7	this work
	230/140 [a]	3.0	1.5	1600 [b]	42.4	3.4	52.4	50.3	43.4	26.6	118.7	this work
**CoMnCs/MC|Rh/POPs-BP&PPh$_3$|CuZrO$_2$/SiO$_2$**	210/140 [a]	3.0	1.5	1600 [b]	24.9	2.2	73.1	72.7	68.5	50.1	111.9	this work
**$Cu_4Fe_1Mg_4$-MMO**	260	3.0	2.0	2400 [b]	39.7	24.4	23.0	18.6 [e]	~13 [e]	-	101	7
**15Co5Fe/AC**	220	3.0	2.0	2800	30.9	1.5	18.1	15.9 [e]	-	5.6 [e]	-	8
**ER-MoS_2-K**	240	5.0	2.0	3000 [b]	17.0	33.6	43.7	30.0 [e]	1.7 [e]	0.0	-	9
**50Mo/50Co**	250	2.0	2.0	15 [c]	2.5	5±2	41.0	18.0 [e]	-	-	-	10
**Cs_2O-Cu/ZnO/Al_2O_3**	280	5.4	3.0	3750	10.0 [d]	20.0 [d]	80.0 [d]	15.0 [d,e]	~5.0 [e]	0.0	-	11
**Co/CuZnO**	250	0.2	2.0	45 [c]	0.1	-	56.9	42.1[e]	-	0.0	-	12
**15Co/SiO_2**	220	3.0	2.0	5600	35.5	-	1.8	-	-	-	-	13
**15Co/AC**	220	3.0	2.0	2800	24.2	0.4	17.5	16.5 [e]	-	9.4 [e]	28	8
**15Co6.3SiO_2/AC**	220	3.0	2.0	500	76.6	1.2	19.9	18.6 [e]	-	10.9 [e]	-	13
**15Co1.9Al_2O_3/AC**	220	3.0	2.0	500	84.9	2.9	18.8	17.9 [e]	-	10.0 [e]	-	13
**15Co/AC-H_2-HNO_3**	210	3.0	2.0	2000	51.5	0.2	10.3	9.2 [e]	-	3.8 [e]	-	14
**15Co0.5Mn/AC**	220	3.0	2.0	2000	40.5	1.8	19.2	17.4 [e]	-	6.1 [e]	50	15
**15Co0.5Mn1La/AC**	220	3.0	2.0	2000	8.3	2.5	26.8	22.9 [e]	-	5.5 [e]	25	16
**15Co2Cr/AC**	220	3.0	2.0	2000			56.9	0.0 [e]	-	0.0	-	17
**15Co0.1Ca/AC**	220	3.0	2.0	900	49.0	0.7	30.6	29.4 [e]	-	16.1 [e]	-	18
**Co/MnO_x@quasi-MOF-74**	230	3.0	2.0	4500 [b]	21.4	0.8	39.0	36.0 [e]	~20 [e]	~14 [e]	18	19
**$Co_1Cu_1Mn_1$**	240	6.0	2.0	2000	18.0	20.0 [d]	37.0	-	-	-	-	1

**Co_2Cu_1**	240	4.0	1.5	2000	5.7	2.7	37.9	27.4 [e]	-	-	-	20
**$Co_1Cu_2Nb_{0.2}$**	200	6.0	1.5	40 [c]	6.0 [d]	1.3 [d]	52.0 [d]	-	-	-	-	3
**$Co_4Mn_1K_{0.1}$**	220	4.0	1.5	40 [c]	7.5 [d]	10.0 [d]	50.0 [d]	~50 [e]	~35 [e]	~22 [e]	44	21
**2.1Ru-CoMn**	220	6.0	1.0	2000 [b]	25.6	30.3	29.9	26.7 [e]	-	-	26	5
**1.1Rh-CoMn**	220	6.0	1.0	2000 [b]	33.1	18.9	33.2	31.7 [e]	-	-	40	5
**CuCoAl│ZnO/ZrO_2**	250	5.0	2.0	4000 [b]	15	-	27.0	20.8 [e]	~8	-	11	22
**CuZnAl-CoMn**	220	6.0	2.0	2000 [b]	12.9	7.3	38.4	36.2 [e]	-	~3	-	23
**CoMn/MAC(P)|Rh/3v-PPh_3@POPs**	200	3	1.5	1250	18.7	8.5	62.1	56.3 [e]	~45 [e]	34.5 [e]	-	24
**CoMn│CuZnAlZr**	220	6.0	2.0	2000 [b]	12.4	6.3	51.1	47.5 [e]	~36 [e]	~33 [d,e]	23	25
**NaPr-CoRu/AomM │Co_2CO_8+PCy_3**	200	1.2	2.0	-	34.0	-	53.7	-	-	1.2 [e]	-	26
**$ZnCrAlO_x$│KNiMoS-MMO-5**	250	5.0	1.0	3000 [b]	10.0	19.0	60.0	44.0 [e]	6.6 [e]	0.0	-	27
**CuCoAl│t-ZrO_2**	-	-	-	-	-	-	64.8	51.6 [e]	-	0.0	-	28

[a] 210 °C (syngas-to-oxygenates/olefins), 140 °C (hydroformylation/hydrogenation).

[b] h^{-1}.

[c] sccm total flow.

[d] estimated value

[e] alcohols and aldehydes.

Table S6. The catalytic performances of different combinations of catalysts in the tandem system for the syngas-to-C_{4+} alcohols conversion.

Catalysts	X_{CO} / %	S / %						Alcohol distribution / C%				
		CH_4	CO_2	C_{2+}	$C_{2+}^{=}$	aldehydes	alcohols	C_1	C_2-C_3	C_4	C_5	C_{6+}
A	15.3	3.7	4.4	17.7	0.5	0.4	73.5	3.1	20.0	18.2	13.0	45.6
B	24.9	3.2	2.2	19.9	1.4	0.0	73.4	0.5	6.4	11.1	14.0	68.6
C	14.7	3.2	1.7	14.0	0.0	0.3	80.8	1.1	8.5	26.3	19.9	44.2

Catalysts	S_{C4+OH} %	S_{C6+OH} %	STY_{OH} g kg_{cat}^{-1} h^{-1}	STY_{C2+OH} g kg_{cat}^{-1} h^{-1}	STY_{C4+OH} g kg_{cat}^{-1} h^{-1}	STY_{C6+OH} g kg_{cat}^{-1} h^{-1}	Distribution of liquid-phase products / wt.%			
							alcohols	aldehydes	olefins	Alkanes
A	56.4	33.7	78.3	76.7	70.8	32.0	93.4	0.2	0.0	6.4
B	68.5	50.0	119.4	118.4	111.9	74.3	89.0	0.2	0.1	10.8
C	73.0	35.7	77.1	72.8	59.2	29.9	95.4	0.3	0.0	4.3

A: Co0.5Mn0.1Cs/C|Rh/3v-POPs-PPh_3|5Pt4Sn/TiO_2.

B: Co0.5Mn0.1Cs/MC|Rh/3v-POPs-BP&PPh_3|$CuZrO_2/SiO_2$.

C: Co0.5Mn0.1Cs/C|Rh/3v-POPs-PPh_3|$CuZrO_2/SiO_2$.

Reaction conditions: H_2/CO = 1.5, F = 130 ml min^{-1}, T = 210 °C (syngas-to-oxygenates/olefins), T = 140 °C (hydroformylation/hydrogenation), and P = 3 MPa.

Table S7. Influence of H_2/CO ratios on the catalytic performances of $Co_{0.5}Mn_{0.1}Cs/C|Rh/3v\text{-}POPs\text{-}PPh_3|CuZrO_2/SiO_2$ tandem system for the syngas-to-C_{4+} alcohols conversion.

H_2/CO mol mol^{-1}	X_{CO} / %	S / %						Alcohol distribution / C%				
		CH_4	CO_2	C_{2+}	$C_{2+}^{=}$	aldehydes	alcohols	C_1	C_2-C_3	C_4	C_5	C_{6+}
1/2	3.7	1.9	3.1	9.9	1.4	1.4	82.2	1.8	6.9	18.5	15.9	56.9
1/1	7.3	2.9	2.2	11.1	0.0	0.2	83.6	2.7	16.1	16.7	13.1	51.4
3/2	14.7	3.2	1.7	14.0	0.0	0.3	80.8	1.1	8.5	26.3	19.9	44.2
2/1	16.0	3.9	1.1	14.4	0.6	0.0	80.0	0.3	12.9	29.0	17.7	40.2
3/1	18.4	4.6	0.8	15.4	1.6	0.0	77.5	2.3	8.5	22.3	17.8	49.2

H_2/CO mol mol^{-1}	S_{C4+OH} %	S_{C6+OH} %	STY_{OH} g kg$_{cat}^{-1}$ h^{-1}	STY_{C2+OH} g kg$_{cat}^{-1}$ h^{-1}	STY_{C4+OH} g kg$_{cat}^{-1}$ h^{-1}	STY_{C6+OH} g kg$_{cat}^{-1}$ h^{-1}	Distribution of liquid-phase products / wt.%			
							alcohols	aldehydes	olefins	Alkanes
1/2	75.0	46.8	31.4	30.2	28.7	14.3	95.0	1.8	0.2	3.3
1/1	67.9	43.0	41.9	39.3	34.0	12.7	97.8	0.3	0.0	1.9
3/2	73.0	35.7	78.3	76.7	70.8	32.0	95.4	0.3	0.0	4.3
2/1	69.5	29.2	68.1	67.8	59.2	23.0	95.3	0.0	0.0	4.7
3/1	69.2	39.1	58.5	55.9	52.2	24.2	95.7	0.0	0.0	4.3

Reaction conditions: F = 130 ml min^{-1}, 210 ^{o}C (syngas-to-oxygenates/olefins), 140 ^{o}C (hydroformylation/hydrogenation), and 3 MPa.

Table S8. Influence of bed temperatures of the syngas-to-oxygenates/olefins reactor on the catalytic performances of $Co_{0.5}Mn_{0.1}Cs/C|Rh/3v\text{-}POPs\text{-}PPh_3|CuZrO_2/SiO_2$ tandem system for the syngas-to-C_{4+} alcohols conversion.

T / ℃	X_{CO} / %	S / %						Alcohol distribution / C%				
		CH_4	CO_2	C_{2+}	$C_{2+}^{=}$	aldehydes	alcohols	C_1	C_2-C_3	C_4	C_5	C_{6+}
210	14.7	3.2	1.7	14.0	0.0	0.3	80.8	1.0	8.5	26.3	19.9	44.2
220 [a]	33.8	5.9	4.3	18.6	1.8	6.9	62.5	1.5	14.1	13.5	11.6	59.3
230	42.4	7.1	3.4	23.4	1.6	12.1	52.4	4.0	13.3	19.7	12.3	50.8
240	72.3	36.3	30.6	18.3	1.4	2.4	11.0	2.6	15.8	24.1	9.1	48.3

T / °C	S_{C4+OH} %	S_{C6+OH} %	STY_{OH} g kg_{cat}^{-1} h^{-1}	STY_{C2+OH} g kg_{cat}^{-1} h^{-1}	STY_{C4+OH} g kg_{cat}^{-1} h^{-1}	STY_{C6+OH} g kg_{cat}^{-1} h^{-1}	Distribution of liquid-phase products / wt.%			
							alcohols	aldehydes	olefins	Alkanes
210	73.0	35.7	78.3	76.7	70.8	32.0	95.4	0.3	0.0	4.3
220	52.8	37.1	128.8	126.9	108.7	76.4	77.3	8.6	0.0	14.0
230	43.4	26.3	143.3	132.6	118.7	60.5	74.8	13.6	0.0	11.6
240	9.0	5.3	50.9	47.9	41.5	14.3	71.4	11.5	0.0	17.1

Reaction conditions: $H_2/CO = 1.5$, $F = 130$ ml min^{-1}, 140 °C (hydroformylation/hydrogenation), and 3 MPa. [a] reaction time: 72 h.

Table S9. Influence of GHSV (based on all catalysts) on the catalytic performances of $Co_{0.5}Mn_{0.1}Cs/C|Rh/3v\text{-}POPs\text{-}PPh_3|CuZrO_2/SiO_2$ tandem system for the syngas-to-C_{4+} alcohols conversion.

GHSV / h^{-1}	X_{CO} / %	S / %						Alcohol distribution / C%				
		CH_4	CO_2	C_{2+}	$C_{2+}^{=}$	aldehydes	alcohols	C_1	C_2-C_3	C_4	C_5	C_{6+}
800 (65 ml min^{-1})	25.8	4.1	1.8	14.8	0.4	0.3	78.7	4.6	9.3	18.8	13.5	53.8
1600 (130 ml min^{-1})	14.7	3.2	1.7	14.0	0.0	0.3	80.8	1.0	8.5	26.3	19.9	44.2
2400 (195 ml min^{-1})	8.5	3.4	1.2	10.0	1.4	0.1	83.8	0.9	8.7	28.6	24.3	37.6
3200 (260 ml min^{-1})	7.3	3.0	0.7	12.5	3.1	0.9	79.8	0.6	5.9	15.3	14.7	63.7
4000 (325 ml min^{-1})	5.9	3.0	0.5	15.3	5.3	2.0	73.9	3.2	15.0	11.6	11.7	58.6

GHSV / h^{-1}	S_{C4+OH} %	S_{C6+OH} %	STY_{OH} g kg_{cat}^{-1} h^{-1}	STY_{C2+OH} g kg_{cat}^{-1} h^{-1}	STY_{C4+OH} g kg_{cat}^{-1} h^{-1}	STY_{C6+OH} g kg_{cat}^{-1} h^{-1}	Distribution of liquid-phase products / wt.%			
							alcohols	aldehydes	olefins	Alkanes
800	67.8	42.3	70.2	63.7	60.6	27.4	95.1	0.4	0.5	4.6
1600	73.0	35.7	78.3	76.7	70.8	32.0	95.4	0.3	0.0	4.3
2400	75.8	31.5	75.7	74.5	68.5	15.7	99.9	0.1	0.0	0
3200	74.8	50.8	76.3	75.4	71.5	43.4	95.4	1.0	0.0	3.6
4000	60.5	43.3	67.2	62.8	55.0	30.2	95.2	1.0	0.0	3.8

Reaction conditions: H_2/CO = 1.5, 210 °C (syngas-to-oxygenates/olefins), 140 °C (hydroformylation/hydrogenation), and 3 MPa.

Table S10. Influence of pressures on the catalytic performances of $Co_{0.5}Mn_{0.1}Cs/C|Rh/3v\text{-}POPs\text{-}PPh_3|CuZrO_2/SiO_2$ tandem system for the syngas-to-C_{4+} alcohols conversion.

P / MPa	X_{CO} / %	*S* / %						Alcohol distribution / C%				
		CH_4	CO_2	C_{2+}	$C_{2+}^{=}$	aldehydes	alcohols	C_1	C_2-C_3	C_4	C_5	C_{6+}
1	4.2	3.8	3.0	15.5	11.4	0.1	66.2	0.3	9.4	12.9	13.4	64.0
2	5.7	3.5	2.3	11.0	4.5	0.1	78.6	0.9	7.5	12.1	15.0	64.5
3	14.7	3.2	1.7	14.0	0.0	0.3	80.8	1.0	8.5	26.3	19.9	44.2
**8**	**17.2**	**1.3**	**1.4**	**10.7**	**0.0**	**0.0**	**86.5**	**0.2**	**7.6**	**22.4**	**17.3**	**52.6**

P/ MPa	S_{C4+OH} %	S_{C6+OH} %	STY_{OH} g kg_{cat}^{-1} h^{-1}	STY_{C2+OH} g kg_{cat}^{-1} h^{-1}	STY_{C4+OH} g kg_{cat}^{-1} h^{-1}	STY_{C6+OH} g kg_{cat}^{-1} h^{-1}	Distribution of liquid-phase products / wt.%			
							alcohols	aldehydes	olefins	Alkanes
1	59.8	41.6	17.8	10.4	16.1	17.7	92.5	0.2	0.0	7.3
2	72.0	50.3	30.1	29.7	27.6	17.6	97.5	0.1	0.0	2.3
3	73.0	35.7	78.3	76.7	70.8	32.0	95.4	0.3	0.0	4.3
**8**	**79.8**	**45.9**	**97.1**	**96.7**	**89.7**	**45.7**	**93.5**	**0.0**	**0.0**	**6.5**

Reaction conditions: $H_2/CO = 1.5$, $F = 130$ ml min^{-1}, 210 °C (syngas-to-oxygenates/olefins), 140 °C (hydroformylation/hydrogenation).

Table S11. Reproduction experiments on the catalytic performances of $Co_{0.5}Mn_{0.1}Cs/C|Rh/3v\text{-}POPs\text{-}PPh_3|CuZrO_2/SiO_2$ tandem system for the syngas-to-C_{4+} alcohols conversion.

Entry	X_{CO} / %	*S* / %						Alcohol distribution / C%					
		CH_4	CO_2	C_{2+}	$C_{2+}^{=}$	aldehydes	alcohols	C_1	C_2	C_3	C_4	C_5	C_{6+}
1	14.9	2.2	1.8	16.9	0.9	0.1	78.1	2.8	4.7	16.7	21.8	17.4	36.6
2	14.2	4.9	1.3	16.5	1.1	0.1	76.2	0.8	4.2	5.8	17.4	16.3	55.5
3	14.7	3.2	1.7	14.0	0.0	0.3	80.8	1.1	3.1	5.4	26.3	19.9	44.2

Reaction conditions: $H_2/CO = 1.5$, $F = 130$ ml min^{-1}, $T = 210$ °C (syngas-to-oxygenates/olefins), $T = 140$ °C (hydroformylation/hydrogenation), and $P = 3$ MPa.

Table S12. Synthetic approach and compositional for catalysts.

Catalyst	Synthesis method	Elements of composition	Nominal Content / wt%	Measured content / wt%
Co/C	incipient wetness impregnation	Co	15	15.2
$Co_{0.5}Mn/C$	incipient wetness impregnation	Co	15	14.9
		Mn	0.5	0.5
$Co_{0.1}Cs/C$	incipient wetness impregnation	Co	15	15.1
		Cs	0.1	0.1
$Co_{0.5}Mn_{0.1}Cs/C$	incipient wetness impregnation	Co	15	14.9
		Mn	0.5	0.5
		Cs	0.1	0.1
Rh/3v-POPs-PPh_3	post-loading	Rh	0.125	0.1
Rh/POPs-BP&PPh_3	post-loading	Rh	0.125	0.1
$CuZrO_2/SiO_2$	ammonia evaporation	Cu	20	19.0
		Zr	-	28.0
Cu/SiO_2	ammonia evaporation	Cu	20	22.0

[a] Determined from ICP-OES.

[b] Determined from XRF.

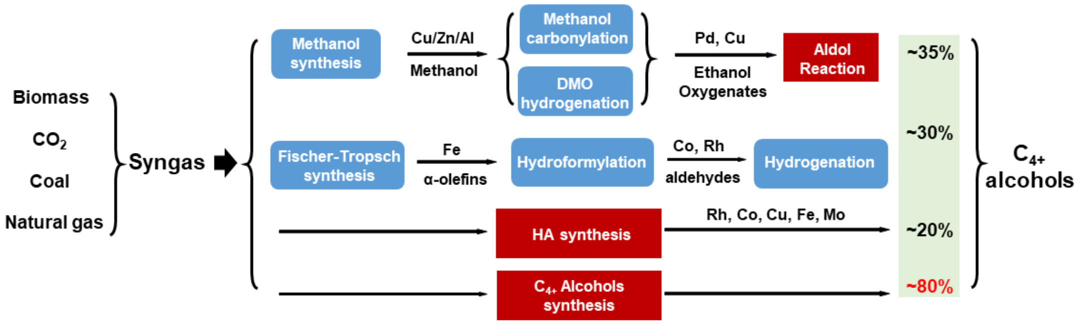

Fig. S1. Different synthetic routes for the production of C_{4+} long-chain alcohols. Commercialized and envisaged processes were highlighted by the blue and red boxes, respectively. Selectivity to C_{4+} alcohols via differen routes was shown in the green box.

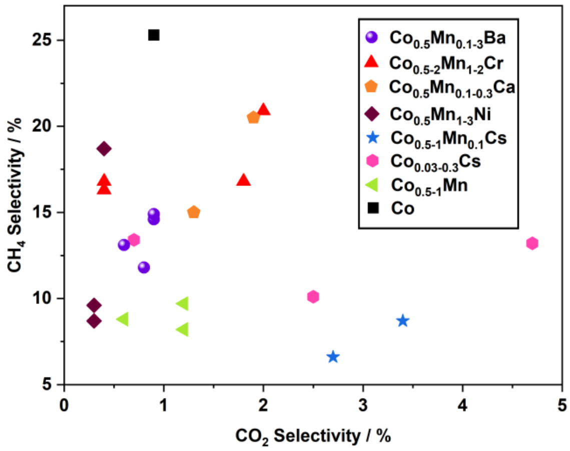

Fig. S2. CO_2 selectivity vs. CH_4 selectivity on carbon-supported Co catalysts with different promoters. (Co_xM_yN: M and N represent metal additives; x and y respectively represent the loading amounts of additives M and N.)

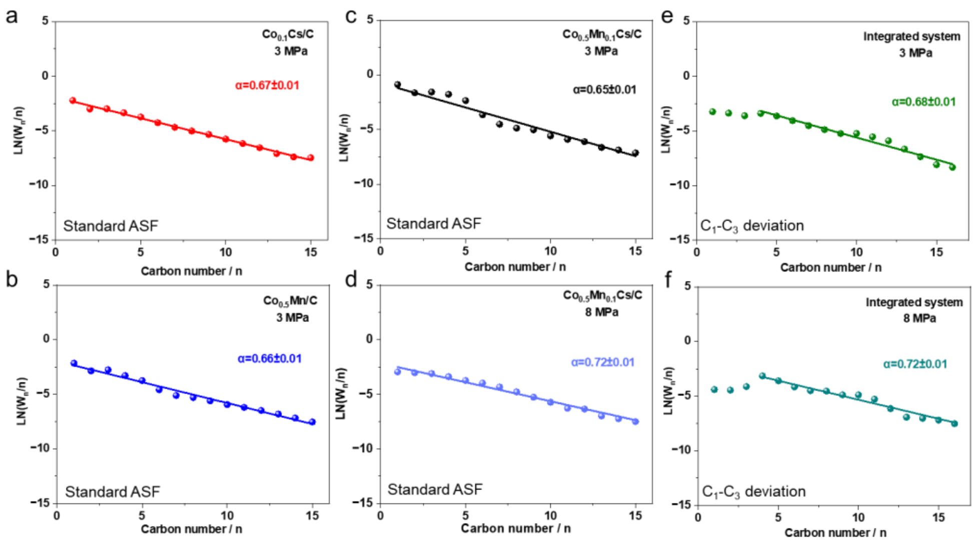

Fig. S3. Products chain lengthening. Detailed selectivity patterns and Anderson-Schulz-Flory (ASF) chain-lengthening characteristics of the various product classes over (a) $Co_{0.1}Cs/C$ (3 MPa), (b) $Co_{0.5}Mn/C$ (3 MPa), (c) $Co_{0.5}Mn_{0.1}Cs/C$ (3 MPa), (d) $Co_{0.5}Mn_{0.1}Cs/C$ (8 MPa). (e) integrated system (3 MPa), (f) integrated system (8 MPa)

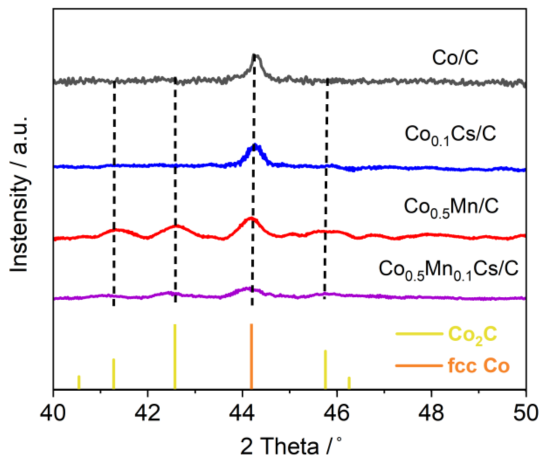

Fig. S4. PXRD patterns of the spent Co_xMn_yCs/C catalysts.

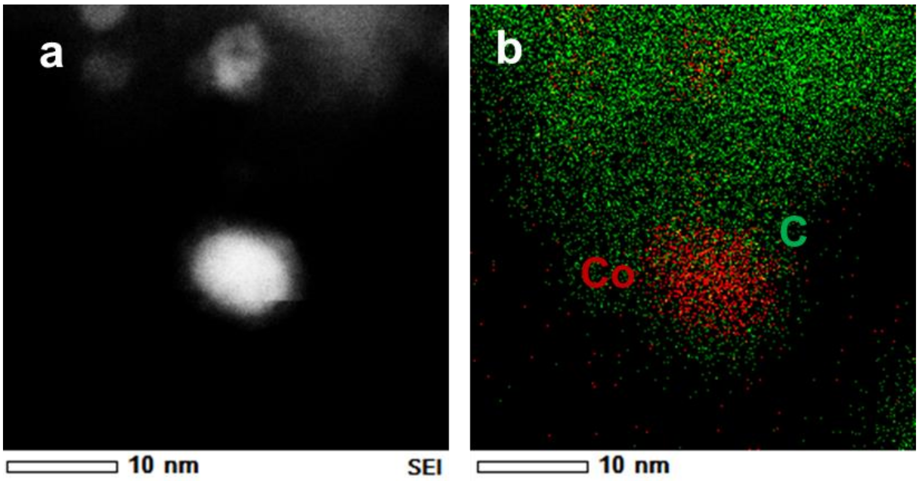

Fig. S5. STEM and EDX mapping images of $Co_{0.5}Mn_{0.1}Cs/C$ catalyst.

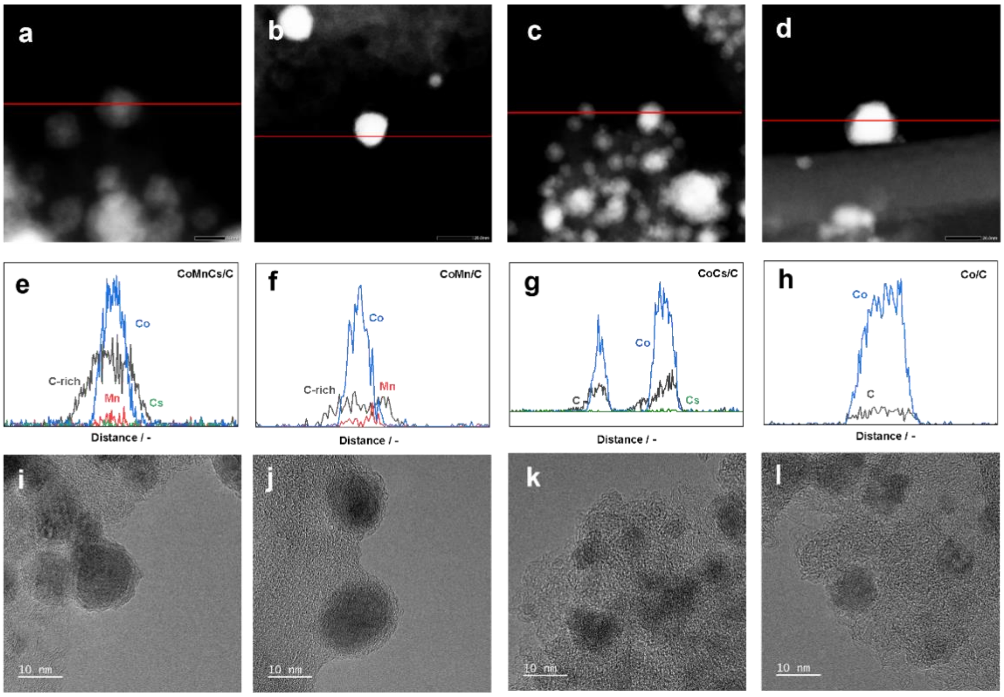

Fig. S6. (a-d) STEM images, (e-g) Line scanning profiles and (i-l) HRTEM images of CoMnCs/C, CoMn/C, CoCs/C and Co/C.

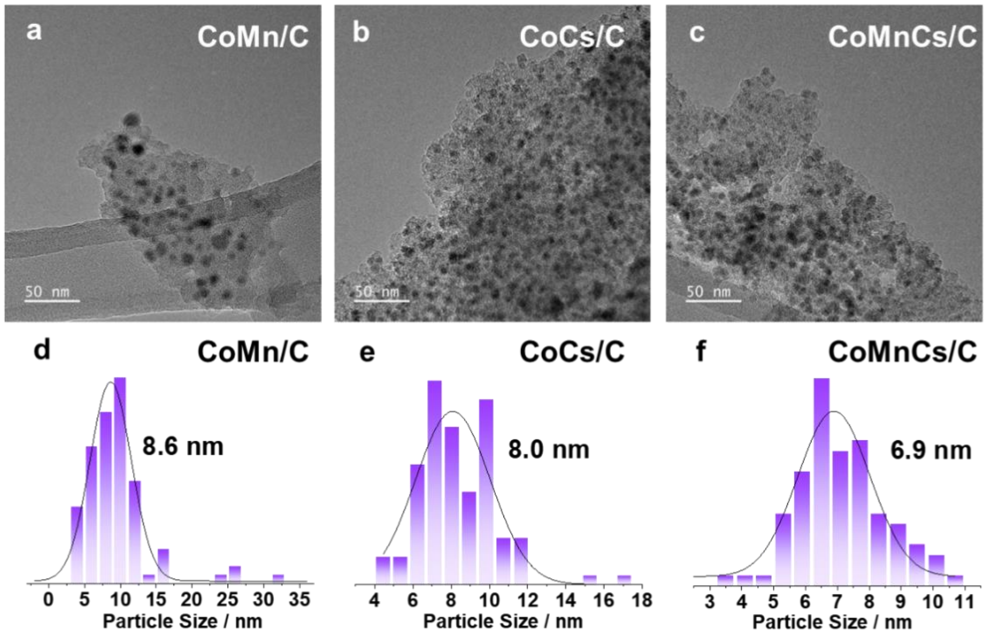

Fig. S7. TEM images of spent $Co_{0.5}Mn_{0.1}Cs/C$ with particle size distributions (inset).

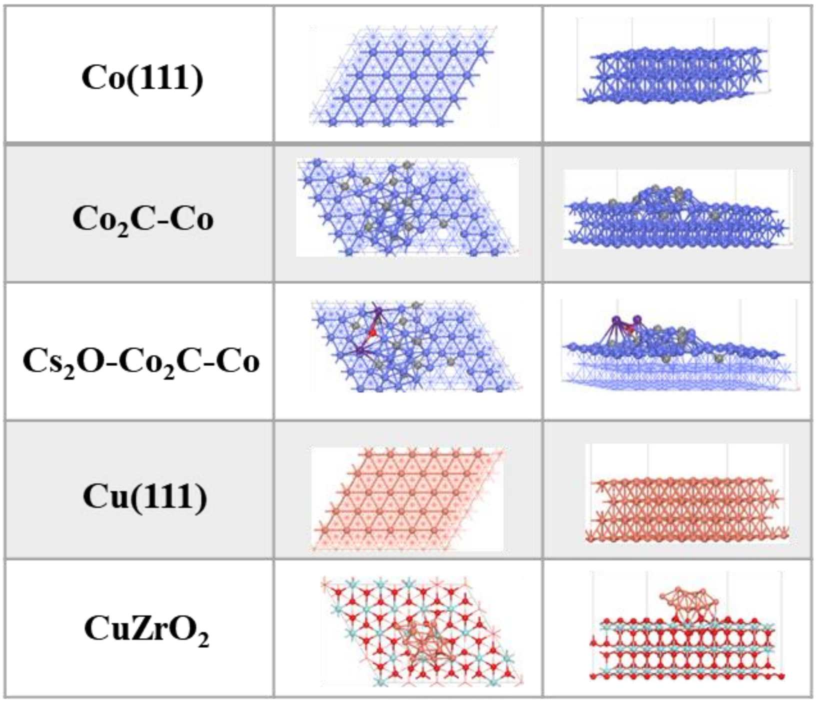

Fig. S8. Illustrations of the top and side views of the surface of Co(111), Co_2C-Co, Cs2O-Co_2C-Co, Cu(111) and $CuZrO_2$.

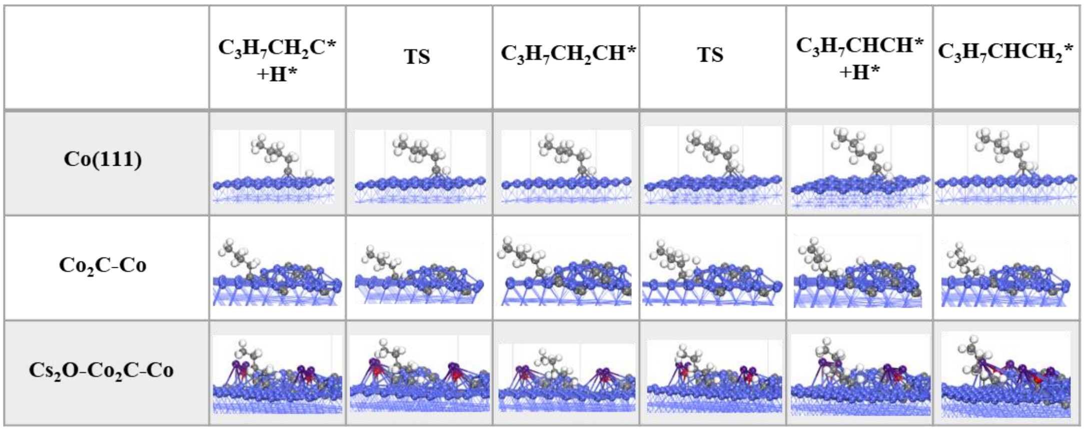

Fig. S9. Illustrations of different configurations of $C_3H_7CH_2C^*$ desorption on the Co(111), Co_2C-Co and Cs_2O-Co_2C-Co.

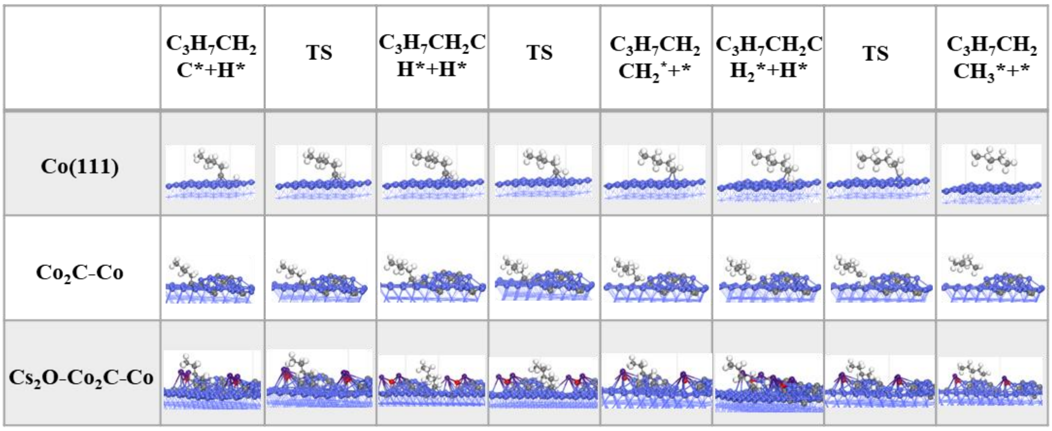

Fig. S10. Illustrations of different configurations of $C_3H_7CH_2C^*$ hydrogenation on the Co(111), Co_2C-Co and Cs_2O-Co_2C-Co.

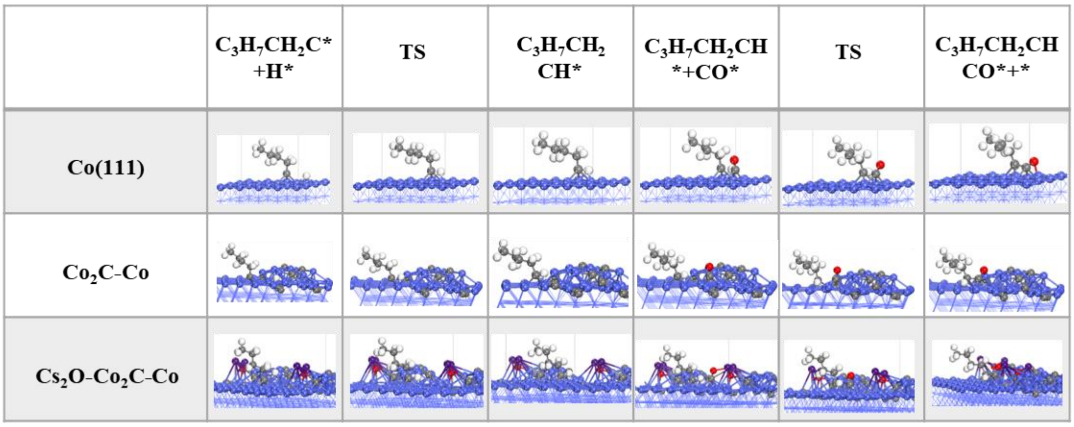

Fig. S11. Illustrations of different configurations of CO* insertion into $C_3H_7CH_2C^*$ species on the Co(111), Co_2C-Co and Cs_2O-Co_2C-Co.

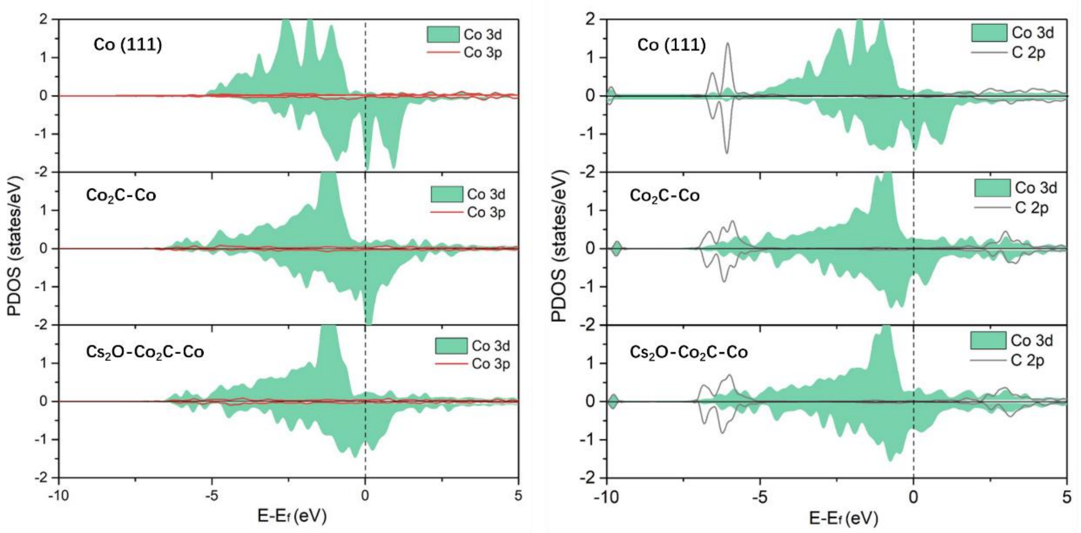

Fig. S12. (a) PDOS for Co(111), Co_2C-Co and Cs_2O-Co_2C-Co (b) PDOS for CO adsorbed on Co(111), Co_2C-Co and Cs_2O-Co_2C-Co.

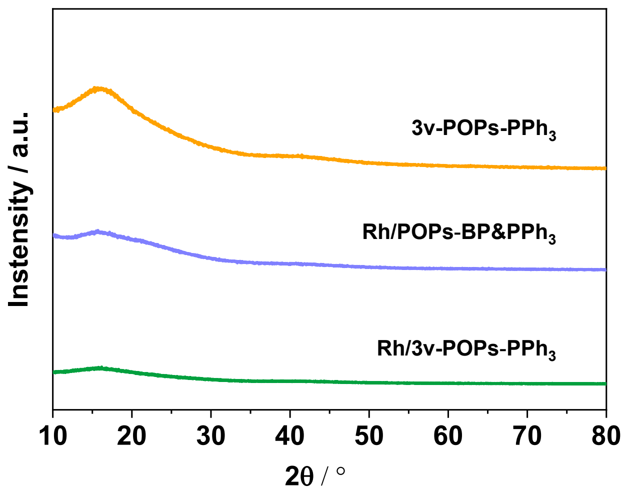

Fig. S13. PXRD patterns of the 3v-POPs-PPh_3 support, spent Rh_1/POPs-BP&PPh_3, and spent Rh_1/3v-POPs-PPh_3 catalysts.

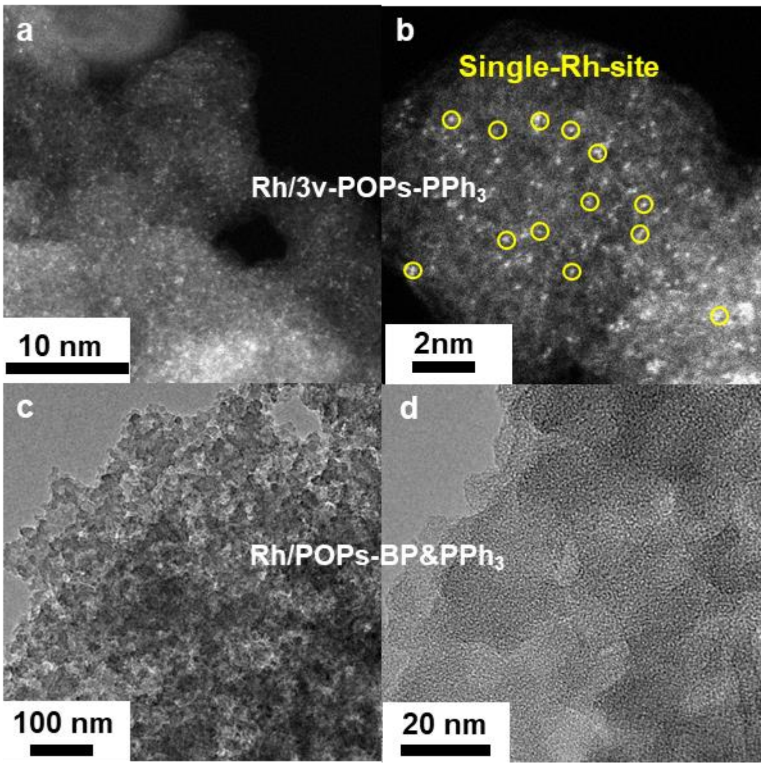

Fig. S14. (a,b) ac-HAADF-STEM images of spent Rh_1/3v-POPs-PPh_3. (c,d) HRTEM images of spent Rh_1/POPs -BP&PPh_3.

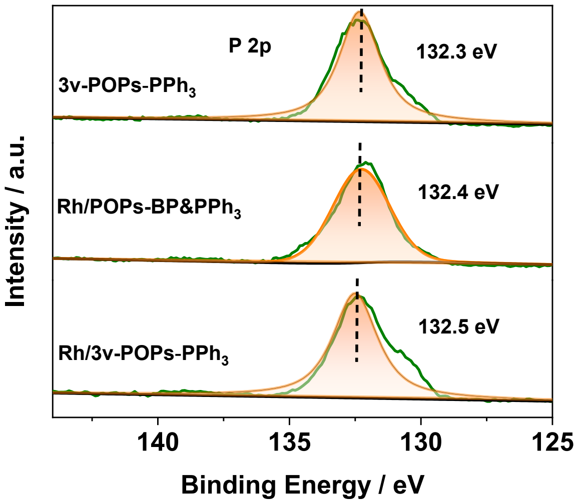

Fig. S15. P 2*p* XPS spectra of the 3v-POPs-PPh_3 support, spent Rh_1/POPs-BP&PPh_3, and spent Rh_1/3v-POPs-PPh_3 catalysts.

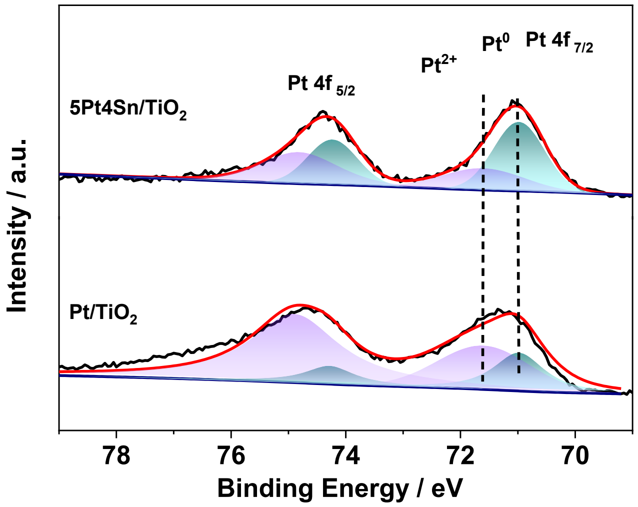

Fig. S16. Pt 4*f* XPS spectra of spent Pt/TiO_2 and spent 5Pt4Sn/TiO_2 catalysts.

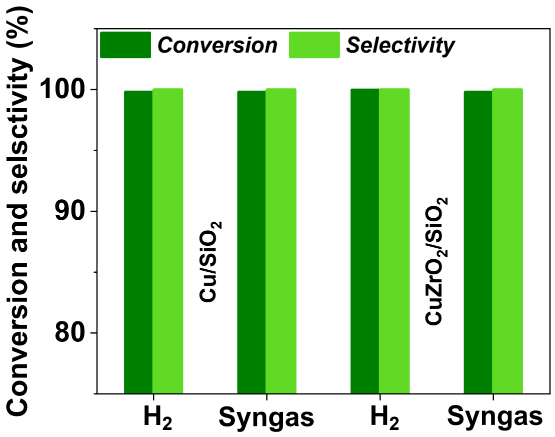

Fig. S17. Catalytic performances of Cu/SiO_2 and $CuZrO_2/SiO_2$ for valeraldehyde hydrogenation in H_2 and syngas streams. Reaction conditions: Cu/SiO_2 and $CuZrO_2/SiO_2$, 1.5 g; P = 3.0 MPa; T = 140 ^{o}C; F (valeraldehyde) = 0.05 ml min^{-1}; F (H_2) or F (H_2/CO = 2) = 60 ml min^{-1}.

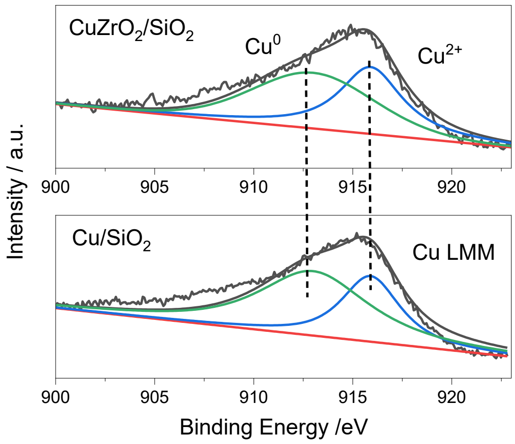

Fig. S18. Cu LMM spectra of the spent Cu/SiO_2 and spent CuZrO_2/SiO_2 catalysts.

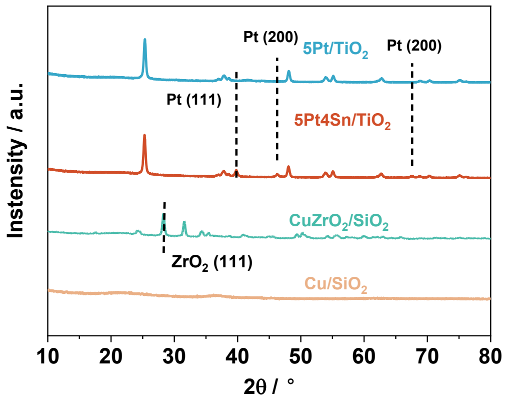

Fig. S19. PXRD patterns of the spent Pt- and Cu-based hydrogenation catalysts.

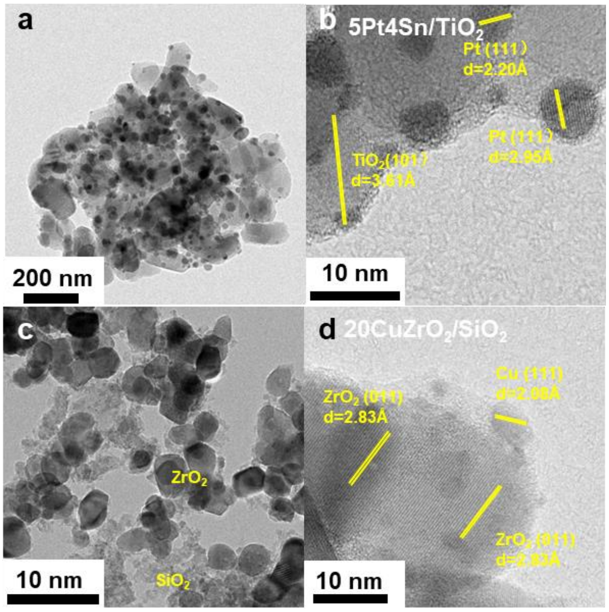

Fig. S20. HRTEM images of spent (a,b) spent 5Pt4Sn/TiO_2 and (c,d) spent $CuZrO_2/SiO_2$.

	$^{*}C_5H_{11}CHO + {}^{*}H$	TS for $^{*}C_5H_{11}CH_2O$	$^{*}C_5H_{11}CH_2O$
**Cu(111)**	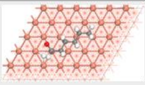	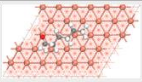	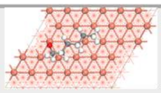
	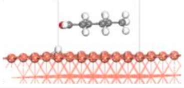	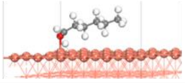	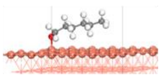
	**$^{*}C_5H_{11}CH_2O + {}^{*}H$**	**TS for $^{*}C_5H_{11}CH_2OH$**	**$^{*}C_5H_{11}CH_2OH$**
	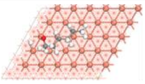	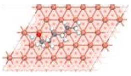	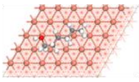
	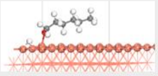	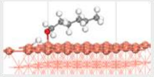	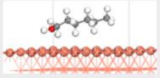

Fig. S21. Illustrations of different configurations of $^{*}C_5H_{11}CHO$ hydrogenation on the Cu(111).

	$^{*}C_5H_{11}CHO + {}^{*}H$	TS for $^{*}C_5H_{11}CH_2O$	$^{*}C_5H_{11}CH_2O$
$CuZrO_2$	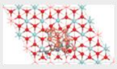	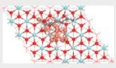	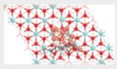
	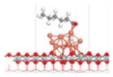	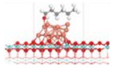	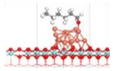
	$^{*}C_5H_{11}CH_2O + {}^{*}H$	TS for $^{*}C_5H_{11}CH_2OH$	$^{*}C_5H_{11}CH_2OH$
	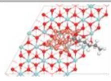	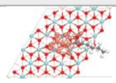	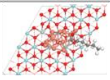
	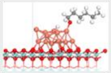	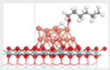	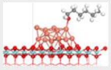

Fig. S22. Illustrations of different configurations of $^{*}C_5H_{11}CHO$ hydrogenation on the $CuZrO_2$.

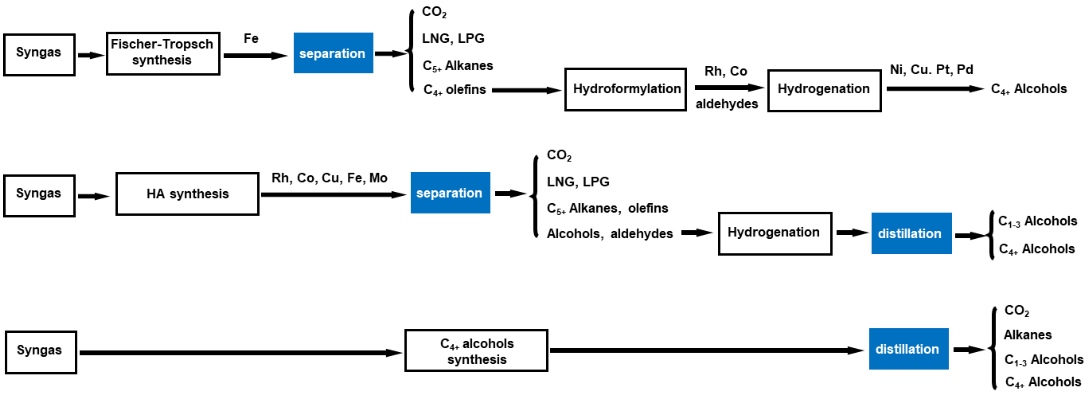

Fig. S23. Different synthetic routes towards C_{4+} alcohols from syngas, accompanied with the corresponding separation and distillation units.

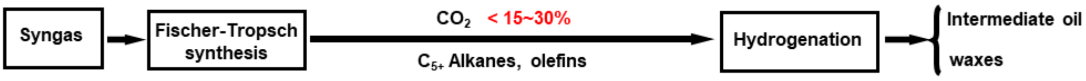

Fig. S24. Fischer-Tropsch synthetic oil process.

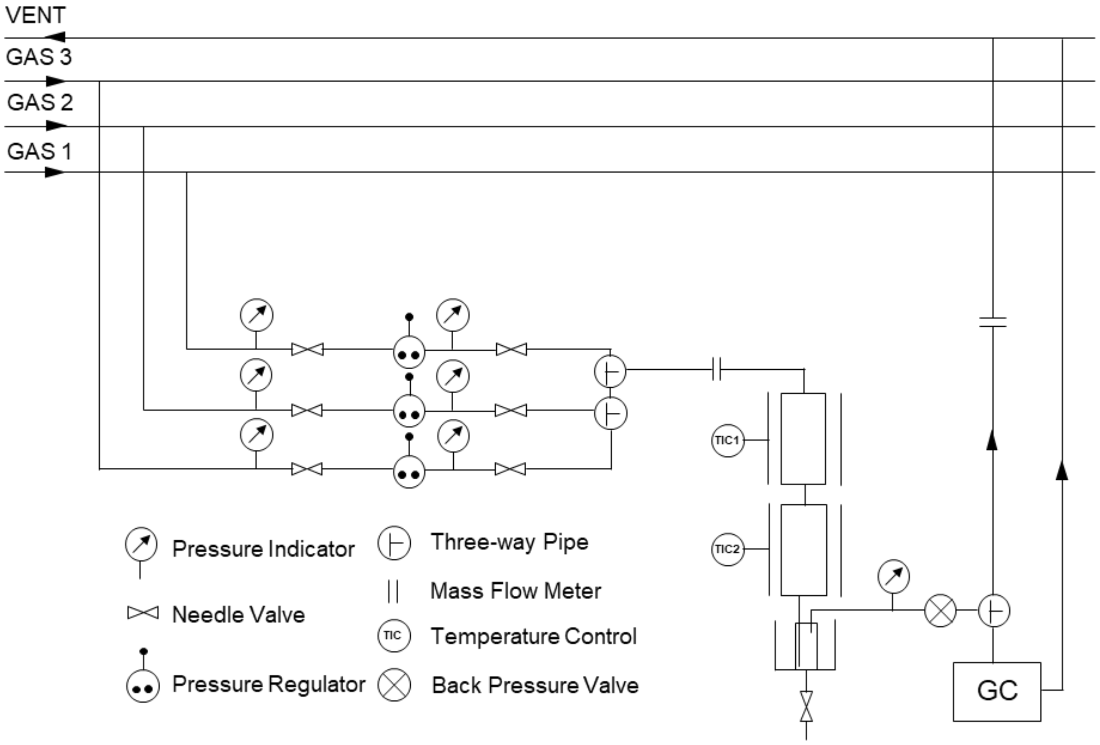

Fig. S25. Process flow diagram of the tandem catalysis system for syngas-to-C_{4+} alcohols reaction.

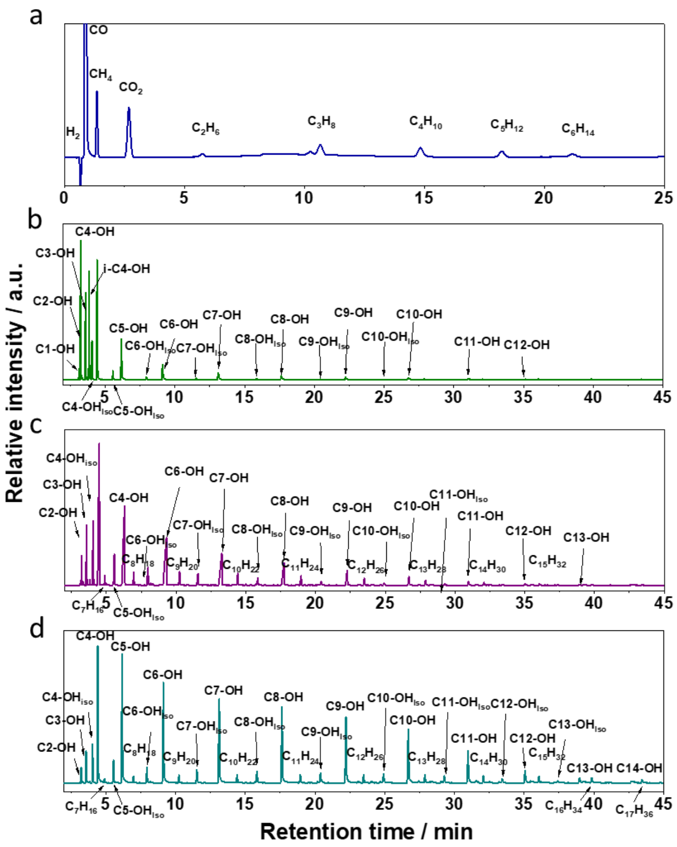

Fig. S26. GC spectra of the (a) gas, (b) water, (c) oil and (d) wax phase products from syngas to C_{4+} alcohols over CoMnCs/C|Rh_1/3v-POPs-PPh_3|$CuZrO_2$/SiO_2.

Supplementary references

1 Xiang, Y., Barbosa, R. & Kruse, N. Higher alcohols through CO hydrogenation over CoCu catalysts: influence of precursor activation. *ACS Catal.* 4, 2792-2800, (2014).

2 Xiang, Y. *et al.* Long-chain terminal alcohols through catalytic CO hydrogenation. *J. Am. Chem. Soc.* 135, 7114-7117, (2013).

3 Xiang, Y., Barbosa, R., Li, X. & Kruse, N. Ternary cobalt–copper–niobium catalysts for the selective CO hydrogenation to higher alcohols. *ACS Catal.* 5, 2929-2934, (2015).

4 Xiang, Y. & Kruse, N. Tuning the catalytic CO hydrogenation to straight- and long-chain aldehydes/alcohols and olefins/paraffins. *Nat. Commun.* 7, 13058, (2016).

5 Qin, T. *et al.* Tuning chemical environment and synergistic relay reaction to promote higher alcohols synthesis via syngas conversion. *Appl. Catal. B: Environ.* 285,119840 (2021).

6 Cui, W. G. *et al.* In situ encapsulated Co/MnOx nanoparticles inside quasi-MOF-74 for the higher alcohols synthesis from syngas. *Appl. Catal. B: Environ.* 278,119262 (2020).

7 Li, Y. *et al.* Interfacial Fe_5C_2-Cu catalysts toward low-pressure syngas conversion to long-chain alcohols. *Nat. Commun.* 11, 61, (2020).

8 Du, H. *et al.* Higher alcohols synthesis via CO hydrogenation on Fe-promoted Co/AC catalysts. *Catal. Today* 281, 549-558, (2017).

9 Hu, J. *et al.* Edge-rich molybdenum disulfide tailors carbon-chain growth for selective hydrogenation of carbon monoxide to higher alcohols. *Nat. Commun.* 14, 6808, (2023).

10 Asundi, A. S. *et al.* Enhanced alcohol production over binary Mo/Co carbide catalysts in syngas conversion. *J. Catal.* 391, 446-458, (2020).

11 Sun, J. *et al.* Promotional effects of cesium promoter on higher alcohol synthesis from syngas over cesium-promoted Cu/ZnO/Al_2O_3 Catalysts. *ACS Catal.* 6, 5771-5785, (2016).

12 Mo, X., Tsai, Y.-T., Gao, J., Mao, D. & Goodwin, J. G. Effect of component interaction on the activity of Co/CuZnO for CO hydrogenation. *J. Catal.* 285, 208-215, (2012).

13 Zhao, Z., Li, Y., Zhu, H., Lyu, Y. & Ding, Y. A review of Co/Co_2C-based catalysts in Fischer–Tropsch synthesis: from fundamental understanding to industrial applications. *Chem. Commun.* 59, 3827-3837, (2023).

14 Li, Y. *et al.* Tuning surface oxygen group concentration of carbon supports to promote Fischer-Tropsch synthesis. *Appl. Catal. A: Gen.* 613, 118017, (2021).

15 Zhao, Z. *et al.* Insight into the formation of Co@Co_2C ctalysts for direct synthesis of higher alcohols and olefins from syngas. *ACS Catal.* 8, 228-241, (2017).

16 Zhao, Z. *et al.* Tuning the Fischer-Tropsch reaction over Co_xMn_yLa/AC catalysts toward alcohols: Effects of La promotion. *J. Catal.* 361, 156-167, (2018).

17 Zhao, Z. *et al.* Increasing the activity and selectivity of Co-based FTS catalysts supported by carbon materials for direct synthesis of clean fuels by the addition of chromium. *J. Catal.* 370, 251-264, (2019).

18 Du, H. *et al.* Study on CaO-promoted Co/AC catalysts for synthesis of higher alcohols from syngas. *Fuel* 182, 42-49, (2016).

19 Cui, W. *et al.* In situ encapsulated Co/MnO_x nanoparticles inside quasi-MOF-74 for the higher alcohols synthesis from syngas. *Appl. Catal. B: Environ.* 278, 119262, (2020).

20 Xiang, Y. *et al.* Long-chain terminal alcohols through catalytic CO hydrogenation. *J. Am. Chem. Soc.* 135, 7114-7117, (2013).

21 Xiang, Y. & Kruse, N. Tuning the catalytic CO hydrogenation to straight- and long-chain aldehydes/alcohols and olefins/paraffins. *Nat. Commun.* 7, (2016).
22 Huang, C., Zhu, C., Zhang, M., Chen, J. & Fang, K. Design of efficient ZnO/ZrO_2 modified CuCoAl catalysts for boosting higher alcohol synthesis in syngas conversion. *Appl. Catal. B: Environ.* 300, d120739 (2022).
23 Wang, J. *et al.* Dihydroxyacetone valorization with high atom efficiency via controlling radical oxidation pathways over natural mineral-inspired catalyst. *Nat. Commun.* 12, (2021).
24 Li, Y. *et al.* Highly selective conversion of syngas to higher oxygenates over tandem catalysts. *ACS Catal.* 11, 14791-14802, (2021).
25 Lin, T. *et al.* Direct production of higher oxygenates by syngas conversion over a multifunctional catalyst. *Angew. Chem. Inter. Ed.* 58, 4627-4631, (2019).
26 Jeske, K. *et al.* Direct conversion of syngas to higher alcohols via tandem integration of Fischer-Tropsch synthesis and reductive hydroformylation. *Angew. Chem. Inter. Ed.* 61, (2022).
27 Luan, X. *et al.* Selective conversion of syngas into higher alcohols via a reaction-coupling strategy on multifunctional relay catalysts. *ACS Catal.* 10, 2419-2430, (2020).
28 Huang, C. *et al.* Direct conversion of syngas to higher alcohols over a CuCoAl|t-ZrO_2 multifunctional catalyst. *ChemCatChem* 13, 3184-3197, (2021).
